# The Effect of Approach/Avoidance Training on Alcohol Consumption Is Mediated by Change in Alcohol Action Tendency

**DOI:** 10.1371/journal.pone.0085855

**Published:** 2014-01-22

**Authors:** Jason M. Sharbanee, Litje Hu, Werner G. K. Stritzke, Reinout W. Wiers, Mike Rinck, Colin MacLeod

**Affiliations:** 1 Centre for the Advancement of Research on Emotion, School of Psychology, University of Western Australia, Crawley, Australia; 2 Department of Clinical Psychology, Behavioural Science Institute, Radboud University Nijmegen, Nijmegen, The Netherlands; 3 Addiction, Development, and Psychopathology (Adapt) Lab, Department of Psychology, University of Amsterdam, Amsterdam, The Netherlands; Cardiff University, United Kingdom

## Abstract

Training people to respond to alcohol images by making avoidance joystick movements can affect subsequent alcohol consumption, and has shown initial efficacy as a treatment adjunct. However, the mechanisms that underlie the training’s efficacy are unknown. The present study aimed to determine 1) whether the training’s effect is mediated by a change in action tendency or a change in selective attention, and 2) whether the training’s effect is moderated by individual differences in working memory capacity (WMC). Three groups of social drinkers (total N = 74) completed either approach-alcohol training, avoid-alcohol training or a sham-training on the Approach-Avoidance Task (AAT). Participants’ WMC was assessed prior to training, while their alcohol-related action tendency and selective attention were assessed before and after the training on the recently developed Selective-Attention/Action Tendency Task (SA/ATT), before finally completing an alcohol taste-test. There was no significant main effect of approach/avoidance training on alcohol consumption during the taste-test. However, there was a significant indirect effect of training on alcohol consumption mediated by a change in action tendency, but no indirect effect mediated by a change in selective attention. There was inconsistent evidence of WMC moderating training efficacy, with moderation found only for the effect of approach-alcohol training on the AAT but not on the SA/ATT. Thus approach/avoidance training affects alcohol consumption specifically by changing the underlying action tendency. Multiple training sessions may be required in order to observe more substantive changes in drinking behaviour.

## Introduction

A defining feature of alcohol addiction is the inability to control consumption, which has been attributed to an imbalance between appetitive and control processes [1]–[4]. This imbalance is thought to arise from a history of alcohol consumption which sensitises the reward system to alcohol-related cues [5]. This *incentive-sensitisation* process manifests as stimulus-driven (or “bottom-up”) biases in two components of the appetitive response [4]–[6]. The first is the relative facilitation of behaviours directed towards alcohol consumption, known as an approach-alcohol action tendency. The second is the selective processing of alcohol-related cues over other stimuli in the environment, known as selective attention to alcohol. Difficulty regulating consumption is thought to arise when these stimulus-driven alcohol biases are disproportionately strong relative to the goal-directed (or “top-down”) control processes.

Alcohol-related biases in both action tendency and selective attention are reliably [7], [8], and independently [9] associated with problem drinking. Further, recent work using training paradigms to manipulate these biases has shown preliminary evidence that the biases causally contribute to addictive behaviour, and suggests the potential clinical application of these training procedures [10], [11].

One of the most promising findings regarding the clinical application of these training paradigms has come from studies training alcohol action tendency [12]–[14] using a variation of the Approach-Avoidance Task (AAT; [15], [16]). In the AAT participants respond to a task-relevant aspect of an image (such as the orientation; landscape or portrait) by moving a joystick which simulates moving the beverage depicted in the image. The joystick movements are accompanied by a zoom-effect which increases the illusion of movement. The premise underpinning the task is that an action tendency elicited by the stimulus will affect response times, such that an approach action tendency will facilitate overt approach-pull movements, and impair overt avoid-push movements. The training variants of the AAT include a contingency, such that the alcohol images are consistently paired with a task requirement to make either an avoidance or an approach response. Thus, an avoid-alcohol training contingency for example, simply requires repeatedly pairing alcohol stimuli with the task-requirement for an avoidance movement.

Such training contingencies have been shown to affect subsequent drinking behaviour, such that heavy drinkers trained with avoid-alcohol contingencies subsequently drink less than those trained with approach-alcohol contingencies [14]. Similarly, alcohol-dependent patients trained with avoid-alcohol contingencies demonstrated less relapse across the twelve months post-treatment than those who received a sham-training [13]. These promising findings, which have since been replicated [12], demonstrate the potential clinical efficacy of AAT training. However, they are not sufficient for the conclusion that the training impacts drinking behaviour through the theoretically specified putative mechanism of changing underlying alcohol action tendency. Evidence for the mechanism of change is important as it provides a basis for optimising of treatment effects and for ensuring that the critical features of the procedure are maintained in clinical practice [17].

### Does AAT Training Work because the Training Contingency Modifies Alcohol Action Tendency?

Several recent reviews have highlighted criteria that should be adopted when seeking to establish the mechanisms of change that underpin treatment effects generally [17]–[19], and for training paradigms specifically [20]–[22]. Two of these criteria are particularly relevant for evaluating the previous training research. First, in order to ensure that any group differences following a training intervention can be attributed to the training contingencies, it is necessary that groups differ only in their exposure to the training contingencies [19], [21], [22]. This can be achieved, for example, by comparing a training group to groups receiving a ‘sham training’ or an opposite training contingency. In contrast comparisons with a ‘no contact’ control condition can not rule out group differences arising from placebo or demand effects, or from differing exposure to the alcohol stimuli rather than the training per se. Second, it is important to statistically verify that the putative mechanism of change mediates the effect of the training on the outcome [17]–[19].

Two of the three studies that have demonstrated an effect of avoid-alcohol training on drinking behaviour have appropriately used a sham-training control condition, but either did not find [13] or did not assess for [14] evidence that the impact of the training on alcohol consumption was mediated by a change in alcohol action tendency. Therefore, while these studies provide evidence that the training-contingency affected alcohol consumption, they do not indicate whether the training worked by changing alcohol action tendency.

Other mechanisms could have been responsible for the change. For example, participants receiving the avoid-alcohol training could have learned to attend less to the alcohol content of the stimuli. This alternative account is plausible since it would have been adaptive in the training context, and because selective attention to alcohol has been shown to be functionally involved in determining drinking behaviour [23], [24]. Therefore, it would be advantageous to evaluate alcohol-related selective attention and action tendency simultaneously, when testing their potential mediating role in alcohol consumption.

One of the three studies that has shown avoid-alcohol training to reduce drinking behaviour used a no-contact control condition, which does not have the capacity to determine whether it was the training contingency that produced the resulting change [12]. This study did show that the impact of training condition on alcohol consumption was mediated by change in alcohol action tendency. Therefore this study provides evidence that training condition was related to change in action tendency, which was also related to clinical outcome. However, it is uncertain whether alcohol action tendency was affected by the training contingency specifically, and not the result of placebo or demand effects, or differing exposure to the stimuli. Together these three studies have shown evidence that avoid-alcohol training can attenuate alcohol consumption, and two of the studies have shown that this cannot be purely attributed to non-specific effects [13], [14]. However, these studies have not demonstrated that the effect of the training is due to the putative causal mechanism.

### Working Memory as a Potential Moderator of Training Efficacy

It is also important to determine potential moderators of training effectiveness, as this could permit identification of those most likely to benefit from the training procedure. We considered working memory capacity (WMC) as a potential moderator of training effectiveness. WMC has been argued to principally reflect attentional control, such that people with high WMC demonstrate less interference from task-irrelevant stimuli (c.f., [25]). Correspondingly, heavy dysregulated drinkers with high WMC demonstrate less interference from task-irrelevant alcohol stimuli on the AAT [26]. Thus, high WMC could also lead to reduced training effects, as processing the task-irrelevant dimension of stimuli (i.e., the alcohol content) is required for any learning from the training-contingencies to take place. Therefore it would be advantageous to evaluate WMC moderation when assessing for mediation of training effects.

### The Present Study

The present study aimed to assess whether the effect of alcohol AAT training on drinking behaviour is mediated by changes in action tendency or selective attention. In order to maximise the chance of observing training effects, we used both approach-alcohol and avoid-alcohol training conditions. The study also used a sham-training control so that the effects of each training conditions could be distinguished. The three groups only differed in their exposure to the alternative training contingencies, in order to determine whether exposure to these training contingencies is specifically responsible for changing alcohol action tendency and alcohol consumption.

Specifically, we assessed whether the impact of AAT training on alcohol consumption was mediated by its impact on alcohol action tendency, rather than by its impact on selective attention to alcohol. This was achieved by first examining the impact of training condition on alcohol-related action tendency and selective attention using a recently developed Selective-Attention/Action-Tendency Task (SA/ATT; [9]), and by then examining the impact of the training conditions and these observed changes in action tendency and selective attention on alcohol consumption observed during a subsequent taste test.

This design incorporates several features recommended for assessing mediation. First it minimises the variance from task-specific learning in the mediation by using a different action tendency assessment task for the mediation, than was used to conduct the training [21], [22]. Second, it permits demonstration of the specificity of the change mechanism, by simultaneously assessing selective attention as another plausible mediator [17], [19]. Third, use of the SA/ATT further reduces method variance confounds, since there are minimal methodological differences between selective attention and action tendency assessment, and the common method variance is partialled out by including both factors simultaneously in the mediation analysis. Finally, we also aimed to assess the potential moderating role of WMC.

When using the AAT and the SA/ATT we have previously observed that approach action tendency and selective attention are less sensitively revealed when facilitating an approach response, than when they interfere with an avoid response [9], [26], [27]. This is likely a ceiling effect arising from the limited ability to speed up an already rapid response, consistent with other measures (e.g., [28]). While the approach-response trials are less sensitive, they also cannot be excluded from the task given a substantial literature across several reaction time paradigms has shown that the degree of interference on incompatible trials (e.g., avoid appetitive) decreases as the proportion of incompatible trials increases [29]–[31]. Therefore we included both approach and avoid response trials, however we expected that the avoid-response trials would show more evidence of an approach action tendency than the approach-response trials.

We predicted that participants exposed to the differing training contingencies would subsequently display differing alcohol action tendency, and drink differing amounts of alcohol during the taste test. If AAT training affects drinking behaviour by changing alcohol action tendency, then the effect of training condition on drinking behaviour will be mediated by change in alcohol action tendency but not by changes in selective attention.

If WMC moderates the training’s effectiveness, there will be WMC by training group interactions, such that lower WMC individuals will show greater action tendency change and greater alcohol consumption than higher WMC individuals will.

## Methods

### Participants

Undergraduate students were eligible to participate if they were over 18, reported drinking beer at least occasionally, and reported weekly alcohol consumption in the middle 50% of a screening sample of 850 candidate participants (i.e., 4 to 22 standard drinks a week). The middle 50% of the drinking distribution was selected in order to allow training in both approach and avoid alcohol directions with reduced risk of ceiling effects. The sample size was determined by the maximum amount of participants that could be recruited within a study period. In total seventy-four participants were recruited, pseudo-randomised to one of the three training conditions, and completed the experimental procedure. The allocation procedure blocked groups of three sequentially tested participants, so that each of the three participants in a block were allocated to a different training condition. This procedure ensured that the three training conditions had equivalent sample size and that testing for the three training conditions was equivalently spaced throughout testing period. The three training groups did not show any significant difference in demographics, on the Alcohol Use Disorders Identification Test (AUDIT), on The Stages of Change Readiness and Treatment Eagerness Scale (SOCRATES), or in WMC (see Table 1). Participants received either course credit, or a $20 reimbursement for their time and effort. All participants gave written informed consent prior to participating, and ethical approval for the study was granted by the University of Western Australia.

**Table 1:** Group descriptives.

	Group			
	Sham-trainingControl	Avoid-alcoholTraining	Approach-alcohol Training	Test Statistic
*Demographics*				
Gender ratio: male:female	8∶16	5∶20	12∶13	χ^2^ (2) = 4.47, *p* = .11
Age: *x¯ (SD)*	19.29 (2.40)	19.48 (2.26)	19.00 (1.53)	*F* (2,73)* = *0.33, *p* = .72
*Questionnaires*	*x¯ (SD)*	*x¯ (SD)*	*x¯ (SD)*	
ACQ (std Drinks/week)	8.14 (6.30)	7.24 (7.87)	10.14 (9.81)	*F* (2,73) = 0.83, *p* = .44
*AUDIT*				
Total	10.13 (4.91)	9.61 (5.21)	9.88 (4.61)	*F* (2,69) = 0.06, *p* = .94
Consumption	5.96 (1.97)	5.96 (2.24)	6.29 (2.29)	*F* (2,71) = 0.19, *p* = .83
Dependence	1.38 (1.61)	1.38 (1.47)	1.24 (1.56)	*F* (2,72) = 0.06, *p* = .94
Problem	2.67 (2.37)	2.50 (2.57)	2.32 (2.12)	*F* (2,72) = 0.13, *p* = .88
*SOCRATES*				
Ambivalence	6.58 (2.65)	6.28 (2.48)	7.76 (3.02)	*F* (2,73) = 2.05, *p* = .14
Recognition	9.46 (2.52)	9.67 (3.40)	10.25 (3.61)	*F* (2,71) = 0.39, *p* = .68
Taking Steps	14.52 (6.35)	13.68 (7.98)	16.88 (7.98)	*F* (2,69) = 1.17, *p* = .32
Operation-Span Score	0.06 (0.96)	0.15 (1.16)	−0.20 (0.87)	*F* (2,73) = 0.83, *p* = .44

Note: ACQ = Alcohol consumption questionnaire; AUDIT = Alcohol Use Disorders Identification Test; SOCRATES = Stages of Change Readiness and Treatment Eagerness Scale. The differences in degrees of freedom for the questionnaire measures are due to skipped items.

### Questionnaires

A brief measure of alcohol consumption was used for the initial screening (Alcohol Consumption Questionnaire, [32], adapted from [33]).We further assessed relevant group characteristics at the time of testing using the AUDIT to assess alcohol consumption, dependence, and alcohol-related problems (which has good reliability and validity, see [34]), and the SOCRATES to assess motivation to reduce alcohol consumption (which has good reliability and validity, see [35]).

### Stimuli

We used the stimuli set from previous experiments using the SA/ATT [9] and this AAT variant [26], [27]. The stimuli consisted of 256 beverage images and 256 abstract images. The beverage image set consisted of 128 alcohol images and 128 non-alcohol images, which were maximally equivalent apart from the presence of alcohol content. There were four different types of alcoholic (beer, wine, spirits, and pre-mixed spirits) and non-alcoholic drink images (soda, juice, coffee, and tea), and there were four different examples of a drink within each of these drink types. Each of these drinks was photographed in unique combination of four different locations (e.g., on an outdoor wooden park table, or on an indoor table with a table cloth), four different actions (e.g., being poured, or being handed towards the camera), and two different glasses or cups.

The 256 abstract images were constructed by cropping small segments of abstract art, selected to contain variations in form and colour, but to be devoid of representations of specific objects. A further 32 images of stationary and office equipment were used for the practice trials.

### Approach-avoidance task (AAT)

The Approach-Avoidance Task was identical to that used by Sharbanee et al. [26], [27] with the addition of a training contingency for the training trials. In contrast to the original AAT [15], where a trial always starts in the middle joystick position, the trials started from an extreme position either with the joystick held maximally close or maximally distal. This task feature prevents error movements on the critical shift trials, consequently the impact of an action tendency can only manifest in the response latencies.

The trials started with an instruction to ‘start distant’ (for an approach-pull trial) or ‘start near’ (for an avoid-push trial). Once this position was held for one second, a single stimulus image appeared on the screen, in minimum zoom for approach-pull trials or in maximum zoom for avoid-push trials, to appear as if the image is distal or proximal from the participant, respectively. The participants then had to respond to the orientation of the picture such that by the end of a trial the images in landscape orientation were close to them, whereas portrait images were away from them. This could involve either moving the joystick (a shift trial), or not responding and keeping the joystick in its original location (a no-response trial).

During the assessment phases, the response latency from the onset of the stimulus until the completion of the movement of the joystick was recorded for each of the approach-pull and avoid-push shift trials. For the no-response trials, no latency was recorded and the participant had to remain in the initial position for one second in order to complete the trial. To encourage participants to respond correctly, a 10-second “time out” was given if the participant moved the joystick on a no-response trial.

Participants first completed 32 practice trials using the office stationary images to learn the task. Once they had demonstrated that they understood the task requirements, they began the main AAT task. The first 64 AAT trials were assessment trials, during which the alcohol and the non-alcohol images appeared equally often in all possible trial types (approach-pull shift, avoid-push shift, start-distant no-response, start-near no-response). The next 384 trials were training trials, and differed according to the assigned training condition. For the approach-alcohol training group, alcohol images were presented only in approach-pull shift trials and start-near no-response trials, and non-alcohol images were presented only in avoid-push trials and start-distant no-response trials. Therefore participants in this training condition were consistently required to respond so that alcohol stimuli ended up appearing maximally proximal. In contrast, for the avoid-alcohol training group, alcohol images were presented only in avoid-push shift and start-distant no-response trials, while non-alcohol images were presented only in approach-pull shift trials and start-near no-response trials. Therefore participants in this condition were consistently required to respond so that alcohol stimuli ended up appearing maximally distal. For the sham-training group, alcohol images and non-alcohol images were presented equally often across all four possible trial types. Regardless of training condition, the final 64 trials of the task were always assessment trials, presenting the alcohol and the non-alcohol images equally often in all possible trial types.

Different subsets of images were used for the training and for the assessment trials, to ensure that any observed effect of the training could be taken to reflect a change in the action tendency for alcohol in general, rather than only a change in the response to the specific stimuli used in the training task. Hence, for each participant, half of the alcohol and non-alcohol images were assigned to training and half to assessment trials. Stimuli assigned to the assessment trials had an equal probability of being assigned to either the pre-training or to the post-training assessment trials. Assignment of stimuli was counterbalanced across participants, such that each image appeared equally often in training trials and in assessment trials.

### Selective Attention/Action Tendency Task

#### Task overview

The Selective Attention/Action Tendency Task (SA/ATT; [9]) measures alcohol-related bias in both selective attention and action tendency. Each trial presented participants with two stimulus images, one beverage image and the other an abstract image. Trials assessing selective attention to alcohol required that participants shifted their focus of attention relative to the beverage images. Trials assessing alcohol action tendency required that participants shifted their hand relative to the beverage image. The selective attention and action tendency trials differ only in the task parameters required to ensure shifting of attentional focus or physical proximity, respectively, and are otherwise equivalent in terms of stimuli, and spatial and temporal parameters.

The task was presented in alternating blocks of selective-attention and action-tendency assessment trials, each block containing 16 trials. A block of each type, using the office equipment images was given in an initial practice. The main task then delivered two blocks each of selective-attention and action-tendency assessment trials. This task was given prior to, and again subsequent to, completion of the AAT. For each participant, the subsets of stimulus images assigned to their pre- and post-training AAT assessment trials were also used for their pre- and post-training SA/ATT assessment trials.

#### Selective attention assessment trials

Each trial commenced with the presentation of two 9 cm^2^ square outlines 165 mm apart on either side of the screen. 500 ms later, a high 500 hz or low 150 hz tone indicated which of these outlines would contain an initial probe. 1300 ms after the tone onset, this probe (a 4 mm line in either horizontal or vertical orientation) was presented in the indicated location for 200 ms. Upon tone offset, one beverage image (50% probability alcohol, and 50% probability non-alcohol) and one abstract image simultaneously appeared, one within each of the two square outlines. 500 ms later a second probe and a foil were presented, one in the locus of each image, simultaneously with the second tone. The second tone instructed the participants to either keep their attentional focus in the original locus (50% of trials) or else shift to the opposing screen location in order to identify the second probe. These latter ‘shift’ trials provided the data of interest, as they enabled assessment of the time taken to make this shift with the differing stimuli. Participants were required to indicate via a mouse button whether the first probe and the second probe were of matching orientation. Thus successful task completion required that attentional focus was sequentially allocated to the locations of the two probes. When the participant’s response was detected the screen was blanked, and the next trial followed after a one second inter-trial interval. Response latency was recorded from the second tone onset until the response was detected. To encourage participants to respond correctly, a 10-second ‘time out’ was given when an incorrect response was made.

#### Action tendency assessment trials

Each trial commenced with presentation of square outlines equivalent to the selective attention assessment. 500 msec later, a high 500 hz or low 150 hz tone indicated which of these screen locations participants had to touch. Participants had to touch the centre of the indicated square region and keep their finger in place until the second tone sounded. Once participants had held their finger down for 200 ms, and at least 1500 ms since the first tone had been presented, one beverage image (50% probability alcohol, and 50% probability non-alcohol) and one abstract image simultaneously appeared, one within each of the two square outlines. 500 ms later the second tone sounded, instructing participants to either lift their finger and retouch the original locus (50% of trials) or else lift their finger and then shift to touch the opposing screen location. These latter ‘shift’ trials provided the data of interest, as they enabled assessment of the time taken to make this shift with the differing stimuli. Thus successful task completion required the participants’ hand sequentially touched the locations indicated by the two successive tones. When the participant’s second touch was detected in the specified region the screen was blanked, and the next trial followed after a one second inter-trial interval. Response latency was recorded from the second tone until the correct touch response was detected. A 10-second ‘time out’ was triggered by premature lifting of the finger before the second tone.

### Calculation of Alcohol-bias Indices

In order to reduce the effect of extreme scores, and consistent with previous research using both the AAT and the SA/ATT [9], [15], [16], [26] median response latencies were used to calculate alcohol-bias indices. The alcohol-bias indices were calculated for both the AAT and the SA/ATT, such that a preference for alcohol was always indicated by a higher score. The presence of an alcohol-related bias will be revealed by faster response times on trials that require an approach response to alcohol, relative to non-alcohol stimuli (i.e., a pull movement on the AAT, or shift from the abstract image to the beverage image on the SA/ATT), or by slower response times on trials that require an avoid response to alcohol, relative to non-alcohol stimuli (i.e., a push movement on the AAT, or shift from beverage image to the abstract image on the SA/ATT). Therefore, alcohol-bias indices for the approach trials were calculated by subtracting the median shift latency for alcohol trials from the non-alcohol trials, so that a positive score reflects facilitated shifting towards alcohol. Alcohol-bias indices for the avoid trials were calculated by subtracting the median shift latency for non-alcohol trials from the alcohol trials, so that a positive score reflects impaired shifting away from alcohol. Therefore, a positive score consistently represents a preference for alcohol relative to non-alcohol.

### Operation-span Task

The operation-span is a WMC task that requires participants to alternate between answering whether simple arithmetic equations are true or false, and reading of a consonant letter to be remembered for subsequent recall (for task details see [36]). The product of the mean memory score and the mean equation accuracy was calculated for each participant to create a composite score that accounts for both letter recall and equation accuracy.

### Taste Test

The taste test procedure from Sharbanee et al. [27] was used. Participants were first asked to consume and rank a selection of drinks given in six shot glasses, three of which were filled with 20 ml of a light beer (below 2.9% alcohol), and the other three with 20 ml of juice. Their preferred beer was subsequently used in the main taste test to ensure that any variation in consumption did not reflect some participants receiving a brand of beer that they disliked. The three beers were accompanied by three shot glasses with 20 ml of juice, so that preference for selecting beer before juice could also be used as a secondary measure of alcohol-consumption motivation (Training-group differences, p = .029, but no significant mediation was observed using this measure. Further details are available on request). Next, participants were required to drink 150 ml of water to prevent the subsequent assessment of alcohol consumption being compromised by variations in thirst.

The main taste test was analogous to previously used taste test designs (e.g., [14]). Participants were provided with a 285 ml glass of their preferred beer, given a taste-rating questionnaire, and were told that the purpose of the task was to rate the beer on several flavour dimensions on the questionnaire. However, the actual purpose of the task was to measure the proportion of the glass of beer that the participant consumed. Participants were told they had to spend a minimum of five minutes filling out the questionnaire, and that they could drink any amount they wished. Participants were not informed of the maximum time limit, but were stopped when they reached ten minutes.

### Procedure

Upon arrival participants were breathalysed to confirm they had a zero blood-alcohol level. The operation-span practice and assessment trials were completed first. The participants then completed the cognitive tasks in the following order: SA/ATT practice trials; SA/ATT pre-training assessment; AAT practice trials; AAT pre-training assessment trials; AAT training trials; AAT post-training assessment trials; SA/ATT post-training assessment trials. The cognitive tasks were followed by the taste test, and then by the questionnaires. Finally, participants were breathalysed again to confirm that their blood-alcohol level was under 0.02. The overall experimental duration was approximately two hours.

## Results

### Overview

**Figure 1:**
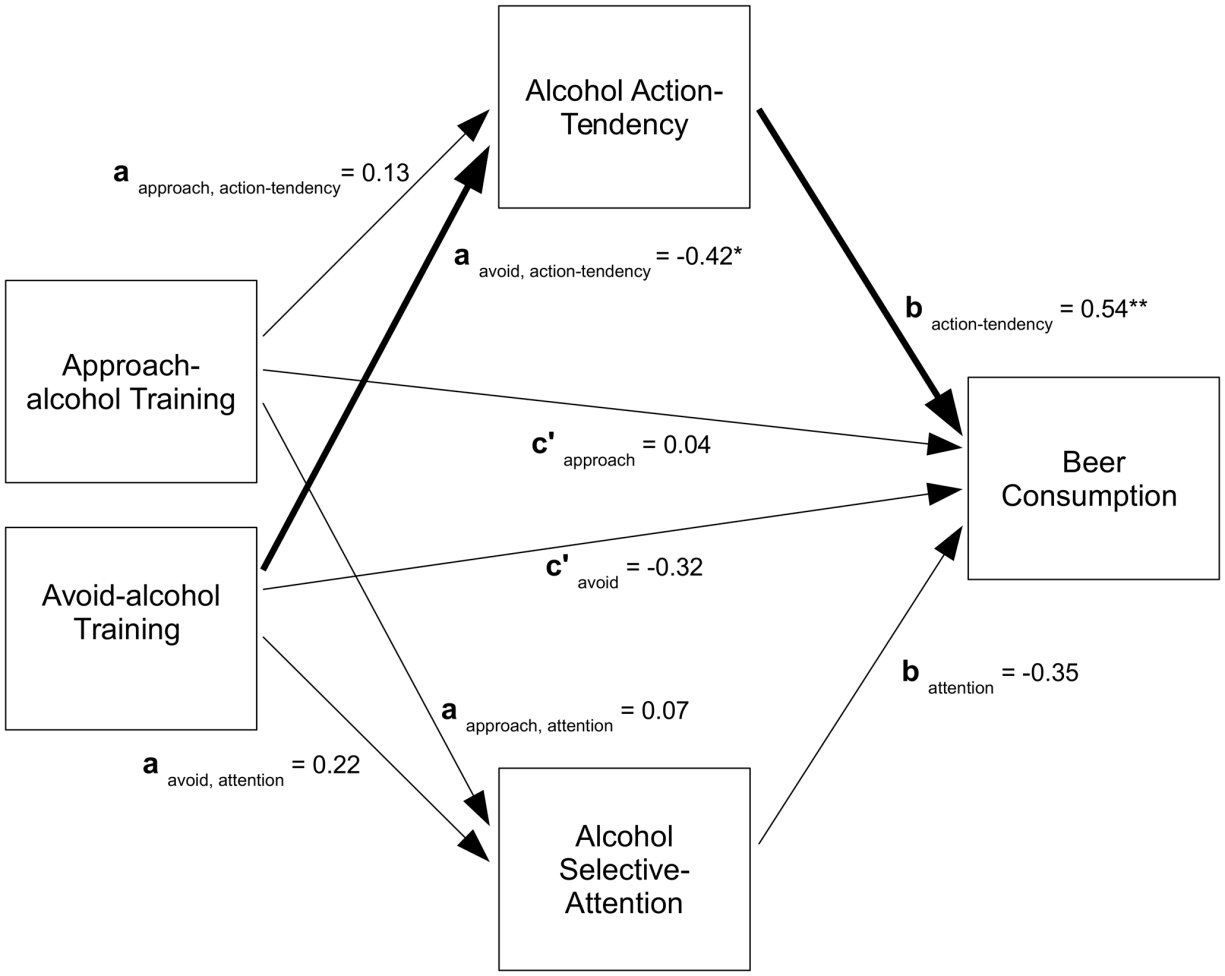
Mediation Diagram. Note that approach-alcohol and avoid-alcohol training variables indicate change relative to the sham-training control. Pre-training alcohol-bias indices are not depicted, but were included in the model as covariates for the relevant paths. The significant paths are indicated in bold.

All data analyses were conducted using R-3.0.2 [37]. We initially addressed whether the AAT training affected participants’ AAT indices, and their subsequent alcohol consumption. We then assessed the mediation of the training following the procedures outlined by Preacher and Hayes ([38], [39],see also [40]). They recommend using a product of coefficients method which quantifies the mediation (i.e., the indirect effect of the independent variable on the outcome variable through the mediator) as the product of the ‘a’ (independent variable – mediator) and ‘b’ (mediator – outcome) paths. This is done in three steps. The first determines the effect of training contingencies on the alcohol-related biases (Figure 1, path a_i_). The second determines the effect of the training contingencies on drinking behaviour after controlling for the mediators (path c’_i_), and the effect of the potential mediators on the drinking behaviour controlling for training groups (path b_i_). The third calculates the indirect effect or mediation as a_i_b_i_.

The training groups were dummy coded, such that each training group variable represents the difference between an active training condition (i.e., approach-alcohol or avoid-alcohol) and the sham-training condition [38]. We also calculated omnibus effects comparing all three training groups, using likelihood-ratio tests to compare models containing both training group dummy variables, to models containing neither of the training group dummy variables.

Prior to analysis, the alcohol-bias indices were screened for outliers following common procedures [41], [42]. Univariate outliers (>3.29 *SD*) were replaced with the next most extreme score in the distribution (0.8% of data). Multivariate outliers were screened in each analysis on the basis of influence, and were removed from the relevant analyses (4.3% of data) if they were separated from the rest of the distribution and exceeded common criteria (i.e., Cook’s D >4/n, or DFFIT >2√(p/n); [41], [43]). Descriptives of the alcohol-bias indices and alcohol consumption are reported in Table 2.

**Table 2:** Alcohol-bias indices, and alcohol consumption for each training group.

	Pre-Training		
Measure	Avoid-Alcohol Training	Sham-Training	Approach-Alcohol Training
	x¯ (SE)	x¯ (SE)	x¯ (SE)
AAT (approach response)	−13.58 (25.09)	−5.88 (44.61)	12.40 (16.69)
AAT (avoid response)	30.44 (27.55)	23.04 (44.91)	15.64 (33.67)
SA/ATT Action-Tendency Index (approach response)	−49.98 (58.14)	−36.06 (87.29)	−52.22 (39.53)
SA/ATT Action-Tendency Index (avoid response)	−34.72 (26.48)	74.15 (47.81)	26.96 (31.96)
SA/ATT Selective-Attention Index (approach response)	−1.14 (44.68)	−20.10 (44.61)	11.30 (55.96)
SA/ATT Selective-Attention Index (avoid response)	30.56 (51.71)	−34.63 (44.91)	−1.54 (59.28)
	**Post-Training**		
**Measure**	**Avoid-Alcohol Training**	**Sham-Training**	**Approach-Alcohol Training**
	**x¯ (SE)**	**x¯ (SE)**	**x¯ (SE)**
AAT (approach response)	−70.74 (16.54)	−7.15 (19.54)	11.54 (24.64)
AAT (avoid response)	−19.62 (25.17)	4.90 (27.24)	30.50 (40.52)
SA/ATT Action-Tendency Index (approach response)	−45.82 (33.26)	−2.63 (61.63)	3.98 (51.66)
SA/ATT Action-Tendency Index (avoid response)	−89.78 (41.73)	−36.69 (83.64)	61.22 (35.20)
SA/ATT Selective-Attention Index (approach response)	33.74 (25.51)	62.85 (46.46)	−79.44 (46.01)
SA/ATT Selective-Attention Index (avoid response)	61.66 (43.23)	−20.98 (60.37)	−23.82 (43.62)
Alcohol Consumption (ml)	115.36 (18.04)	123.96 (19.43)	150.44 (16.99)

### Impact of AAT Training on AAT Indices

The data showed evidence of heteroscedasticity across the training groups, therefore we used linear-mixed models in order to accommodate both the heteroscedasticity and the dependency across the repeated-measures covariates. On the basis of likelihood-ratio tests, the models used a covariance matrix with correlated residuals and heterogenous variance across the training groups, and no random coefficients. All comparisons between models are based on maximum likelihood estimations, whereas the significance of each predictor is based on restricted maximum likelihood estimations.

In order to assess whether the training contingency successfully altered action tendency towards alcohol, a linear-mixed model was run on the post-training AAT index, with training group, approach/avoid trial type, pre-training AAT index and their interactions as independent variables. There were no significant interactions involving approach/avoid trial type, or involving pre-training AAT indices *p*s >.119, which were removed from subsequent models. There was also no significant effect of pre-training AAT index, *b* = −0.03, 95% CI [−0.18,0.11], *p = *.645, or approach/avoid trial type, *b* = −0.03, 95% CI [−0.16,0.10], *p = *.612. More importantly, there was an omnibus main effect of training group, χ^2^(2) = 6.87, *p* = .032, with both training groups showing changes relative to the sham-training group in the expected direction. However, these effects did not reach significance when the approach-alcohol, *b = *0.26, 95% CI [−0.19,0.72], *p = *.263, or the avoid-alcohol groups, *b* = −0.30, 95% CI [−0.66,0.05], *p = *.096, were considered individually.

We then assessed for WMC moderation by adding WMC and related interactions to the model. There was no main effect of WMC, *b = *0.12, 95% CI [−0.15,0.39], *p = *.385. But more importantly, there was a significant interaction between the approach-alcohol training and WMC, *b* = −0.51, 95% CI [−1.01,0.00], *p = *.048, but not between the avoid-alcohol training and WMC, *b* = −0.06, 95% CI [−0.41,0.29], *p = *.745. The interaction in the approach-alcohol group was such that the difference between the approach-alcohol group and the sham group was greater at lower levels of WMC, consistent with the interpretation that lower WMC would have greater training effects. Thus, the hypothesis that the training would affect the AAT indices was supported and the hypothesis that WMC would moderate the training efficacy was supported for the approach-training group only.

### Impact of AAT Training on Alcohol Consumption

Next we tested the effect of the training contingencies on drinking behaviour using a generalised-linear model with quasi-binomial errors and a logit link, since the dependent variable is a proportion [44]. The generalised-linear model was run on the proportion of alcohol consumption, with the training groups as the independent variable., Both training groups showed a trend towards changing in the expected direction relative to the sham-training group. However, this omnibus main effect of training group approached, but did not reach significance, χ^2^ (2) = 2.03, *p* = .053, and these effects were not significant when the approach-alcohol, *b = *0.47, 95% CI [−0.24,1.16], *p = *.173, or the avoid-alcohol groups, *b* = −0.37, 95% CI [−1.10,0.33], *p = *.312, were considered individually.

### Mediation Analysis

Consistent with previous findings, preliminary analyses suggested that the approach-response trials may not have been sufficiently sensitive to individual differences in alcohol related biases in the present SA/ATT data. In contrast with the avoid-response trial indices (see below), the approach-response trial indices were not significantly related to quantity of alcohol consumption, b_action tendency_, *p* = .084, b_selective attention_, *p* = .492, nor did they correlate with the indices from the avoid trials, *r*s<|.13|, *p*s >.29. Consequently, we concluded that the approach-response trials may not be sufficiently sensitive, and the following mediation analyses were conducted using the indices from the SA/ATT avoid-response trials only.

Since testing indirect effects can violate the assumption of normality, bootstrapping was used to calculate bias-corrected accelerated confidence intervals [39], and permutation tests were used to derive the *p* values for all analyses (using the permute-residuals method, and 5000 resamples; [40], [45]). The paths of the mediation analysis are shown in figure 1, with the significant paths in bold.

#### Effect of AAT training on SA/ATT measure of action tendency (path a_action tendency_)

We tested whether the AAT training impacted upon the SA/ATT measure of action tendency, by running a linear model on the post-training SA/ATT action tendency index, with the training groups and the pre-training SA/ATT action tendency index as the independent variables.

There was no significant effect of the pre-training index, *B* = −0.07, 95% CI [−0.26,0.05], *p* = .276. More importantly, the omnibus test of training showed that the training groups significantly differed in their post-training action tendency index, χ^2^ (2) = 4.06, *p* = .009. As can be seen in Figure 1, the avoid-alcohol group showed significantly less action tendency index than the sham-training group, *a*_avoid, action tendency_ = −0.42, 95% CI [−0.88, −0.10], *p* = .032, and the approach-alcohol group showed non-significantly greater action tendency index than the sham-training group, *a*_approach, action tendency_ = 0.13, 95% CI [−0.23,0.45], *p* = .481. Hence, the hypothesis that the AAT training would affect alcohol action tendency was supported, and this effect was mainly carried by the avoid-alcohol group demonstrating less action tendency towards alcohol than the sham-training group.

#### Effect of AAT training on SA/ATT measure of selective attention (path a_selective attention_)

To test the AAT training impact on the SA/ATT measure of selective attention to alcohol cues, we ran a linear model on the post-training SA/ATT selective attention index, with the training groups and the pre-training SA/ATT selective attention index as independent variables. There was no significant effect of the pre-training index, *B* = −0.03, *p* = .276. More importantly, there were no significant training-group differences overall, χ^2^ (2) = 0.57, *p* = .602, or for either the approach-alcohol group, *a*_approach,selective attention_ = 0.07, 95% CI [−0.37,0.52], *p* = .737, or avoid-alcohol group specifically, *a*_avoid,selective attention_ = 0.22, 95% CI [−0.22,0.66], *p* = .336. Therefore, there is no evidence that the AAT training affected selective attention to alcohol cues.

#### Effect of AAT training (path c’) and alcohol-related biases (path b) on alcohol consumption

Next we assessed the relative-direct effect of the training groups (path c’) and the mediator effects (path b) on alcohol consumption, using a generalised linear model with quasi-binomial errors and a logit link. We ran the generalised linear model on the quantity of alcohol consumption, with the training groups, and the pre and post training SA/ATT indices for both action tendency and selective attention as the independent variables. There was no significant effect of the pre-training selective attention index, *B* = −0.11, *p* = .498. However, there was a significant effect of pre-training action tendency index, *B* = −0.36, *p* = .034, indicating that those with lower action tendency before the training, drank more in the taste test. There was no significant effect of training group on drinking behaviour once the variance from the action tendency and selective attention had been partialled out, χ^2^ (2) = 0.38, *p* = .325, and this was consistent across both the approach-alcohol training group, *c’*_approach_ = 0.04, 95% CI [−0.81,0.87], *p* = .919, and the avoid-alcohol training group, *c’*_avoid_ = −0.32, 95% CI [−1.16,0.47], *p* = .416. This indicates that there was no effect from the training that was not accounted for by the variance in the action tendency and selective attention indices.

The post-training selective attention index also showed no significant relationship with alcohol consumption in the taste test, *b*_selective attention_ = −0.35, 95% CI [−0.73,0.08], *p* = .067. Further, the direction of this non-significant association was opposite to what would be expected if changes in selective attention affected alcohol consumption. In contrast, greater post-training action tendency index was associated with greater alcohol consumption, *b*_action tendency_ = 0.54, 95% CI [0.17,0.89], *p = *0.009, consistent with the hypothesis that alcohol action tendency affects alcohol consumption.

#### The indirect effect of AAT training through the mediatiors (ab)

To calculate the indirect effect, that is the effect of the training contingencies through the mediators, we calculated the product of the effect of training on the mediators (paths a_i_) and the effect of the mediators on alcohol consumption (paths b_i_). The permutation test for this effect is based on a reduced model with both a and b paths equal to zero. There was a significant indirect effect of the avoid-alcohol training (relative to the sham-training) on alcohol consumption through action tendency, *ab*_avoid-action-tendency_ = −0.23, 95% CI [−0.59, −0.04], *p*<.001, but not through attention, *ab*_avoid-attention_ = −0.03, 95% CI [−0.29,0.17], *p* = .249, and there was a significant difference between these pathways, *p* = .001. Similarly, there was a significant indirect effect of approach-alcohol training (relative to the sham-training), through the action tendency, *ab*_approach,action tendency_ = 0.07, 95% CI [−0.14,0.29], *p* = .033, but not through selective attention, *ab*_approach-attention_ = −0.01, 95% CI [−0.21,0.23], *p* = .602, however the difference between these pathways was not significant, *p* = .074. These findings are consistent with the specificity of the action tendency mediation.

#### Assessment of WMC moderation

Finally we examined if the effect of the training was moderated by WMC, by adding WMC and the associated interactions to the models. There was no evidence of WMC moderating the effect of training on action tendency, χ^2^ (2) = 0.69, *p* = .291, or on selective attention, χ^2^ (2) = 0.31, *p* = .731. Similarly, there was no evidence that WMC moderated the effect of training on alcohol consumption independent of the action tendency and selective attention paths, χ^2^ (2) = 0.26, *p* = .762. Further, as WMC was unrelated to the action tendency or selective attention indices, it suggests that differing WMC demands cannot account for the differences between the action tendency and selective attention pathways.

## Discussion

The present study sought to replicate the previous finding that AAT training can alter subsequent alcohol consumption, while extending understanding of this effect by testing whether it is carried by modification of alcohol action tendency. The study successfully induced biased responses on the AAT consistent with the training conditions, however there was no significant main effect of the training conditions on subsequent alcohol consumption in the taste test. Most importantly, avoid-alcohol AAT training influenced a subsequent measure of alcohol action tendency, but not a measure of selective attention to alcohol cues, and there was a significant indirect effect of the training condition on alcohol consumption that was mediated by this action tendency path.

While the demonstration of action tendency mediation is consistent with the findings of Eberl et al. [12], the present results also extend the findings of Eberl et al. [12] in several ways. Importantly, as far as we are aware this is the first evidence of mediation using comparison groups that only differ in their exposure to the training contingencies that has been reported for training any alcohol-related bias, including attentional and interpretation training. Several theorists have emphasised the importance of demonstrating mediation by the putative mechanism in establishing a potential treatment technique, something that is rarely assessed in psychological research [17]. Further, as this study is the first to show the mediation while using comparison groups that only vary in the exposure to the training contingencies, it provides the first evidence that the training contingencies are specifically responsible for the mediated effects. These results therefore take an important step in validating this approach, both as a means of experimentally manipulating these processes to assess causality, and as a potential treatment or treatment adjunct.

As well as demonstrating mediation through the action-tendency pathway, we also assessed the potential mediation through the selective attention pathway. There was no evidence of the training contingencies affecting alcohol-related bias in selective attention. This provides some evidence against the possibility that the training contingencies were actually working through this alternative mechanism, although it does not preclude the possibility that an effect was not observed due to insufficient power or an insufficient dose of training. Further, since there was no change in the methodologically similar selective attention assessment, and as action tendency was assessed on a different task to the training task, it is unlikely that the difference between the training groups in action tendency was due to task-specific learning (i.e. method variance) rather than change in action tendency per se.

The present findings showed no evidence that post-training selective attention to alcohol was related to alcohol consumption, unlike previous findings [7], including findings from one study that used the same SA/ATT assessment methodology [9]. This suggests that alcohol consumption was driven more by the action tendency than by selective attention immediately post-training, whereas both processes may make a more equal contribution to drinking behaviour in other situations (e.g., [9]).

There was some partial support for the role of WMC as a moderator of training efficacy, since WMC moderated the post-training responses on the AAT for the approach training group, but not the avoid training group. However, this moderation did not generalise to the SA/ATT measure of action tendency, thus showing inconsistent support for the moderating role of WMC. Other studies have assessed for training moderation using the Stroop task as a measure of top-down control, and have also found inconsistent results with one study reporting the moderation [46] and one failing to find it [12]. Determining whether this inconsistency is due to the effect being present but small, or whether it is spurious will require further research. However, in both the present finding and the finding of Salemink et al. [12] the direction of the effect was consistent with theoretical expectations, adding some weight to the former possibility.

The notable weakness in the present findings is that the main effect of the training on alcohol consumption was not statistically significant. The relatively modest and non-significant difference in drinking behaviour (35 mls or 12% of the glass) is consistent with a previous finding that also assessed the effect of a single training session on a student population [14]. Together these findings suggest that a single session of approach/avoidance training is not sufficient to produce substantive changes in student’s drinking behaviour.

These findings contrast with the more impressive extent of behaviour change observed in studies that have used multiple training sessions with clinical populations [12], [13]. The contrast is likely to be partly due to the different populations tested, given moderation analyses have shown that avoid-alcohol training is more effective with participants who have more severe alcohol use or problems (indicated by the amount of previous detoxes, 12,or weekly alcohol consumption, [14]). It is also likely that the increased amount of training sessions used in the Wiers et al. [13] and Eberl et al. [12] studies also increased the training effect.

While the group differences in alcohol consumption were not significant, it should be noted that the mediation is not dependent on the presence of this effect (c.f., [47], [48]). Thus, the current findings provide the first evidence that approach-alcohol action tendency mediates the influence of AAT training on alcohol consumption, while using comparison conditions that only vary in the training contingency. Hence, this study sheds valuable light on the mechanisms that underpin the therapeutic efficacy of AAT training.
